# Fundamental problems with the cooperative breeding hypothesis. A reply to Burkart & van Schaik

**DOI:** 10.1111/jzo.12351

**Published:** 2016-05-24

**Authors:** A. Thornton, K. McAuliffe, S. R. X. Dall, E. Fernandez‐Duque, P. A. Garber, A. J. Young

**Affiliations:** ^1^ Centre for Ecology and ConservationUniversity of ExeterPenrynUK; ^2^ Department of PsychologyYale UniversityNew HavenCTUSA; ^3^ Department of PsychologyBoston CollegeChestnut HillMAUSA; ^4^ Department of AnthropologyYale UniversityNew HavenCTUSA; ^5^ Department of AnthropologyUniversity of IllinoisUrbanaILUSA

**Keywords:** cooperative breeding hypothesis, callitrichid monkeys, evolution, prosociality, social learning, social tolerance, teaching

## Abstract

The cooperative breeding hypothesis (CBH) states that cooperative breeding, a social system in which group members help to rear offspring that are not their own, has important socio‐cognitive consequences. Thornton & McAuliffe (2015; henceforth T&M) critiqued this idea on both conceptual and empirical grounds, arguing that there is no reason to predict that cooperative breeding should favour the evolution of enhanced social cognition or larger brains, nor any clear evidence that it does. In response to this critique, Burkart & van Schaik (2016 henceforth B&vS) attempt to clarify the causal logic of the CBH, revisit the data and raise the possibility that the hypothesis may only apply to primates. They concede that cooperative breeding is unlikely to generate selection pressures for enhanced socio‐cognitive abilities, but argue instead that the CBH operates purely through cooperative breeding reducing social or energetic constraints. Here, we argue that this revised hypothesis is also untenable because: (1) it cannot explain why resources so released would be allocated to cognitive traits *per se* rather than any other fitness‐related traits, (2) key assumptions are inconsistent with available evidence and (3) ambiguity regarding the predictions leaves it unclear what evidence would be required to falsify it. Ultimately, the absence of any compelling evidence that cooperative breeding is associated with elevated cognitive ability or large brains (indeed data suggest the opposite is true in non‐human primates) also casts doubt on the capacity of the CBH to explain variation in cognitive traits.

## Introduction

Part of the difficulty in assessing the CBH is that its causal logic has been presented in two distinct forms. At times, the arguments are presented in the language of natural selection and adaptation. For example Burkart & van Schaik (2010) suggest that wolves have ‘socio‐cognitive adaptations to cooperative breeding’ (p. 12) and discuss ‘the selection pressures associated with extensive allomaternal care’ (p. 14). This seems to frame the CBH as an adaptive hypothesis, similar to the social intelligence or social brain hypotheses (Humphrey, 1976; Dunbar, 1998), positing that cooperative breeding generates selection for enhanced socio‐cognitive abilities. In their response to our critique, B&vS agree that such an adaptive argument is untenable: ‘we can only agree with T&M's conclusion that “there is no evidence that [the cognitive and motivational processes found in cooperative breeders] are either unique to cooperative breeders or particularly cognitively demanding” and “that there is little evidence to suggest that cooperative breeding entails distinct cognitive challenges”’. If cooperative breeding does not generate novel selection pressures on cognitive processes, then it follows that there are no novel benefits for enhanced cognition or large brains in cooperative breeders. What then does the CBH have to offer?

The more common version of the CBH (which B&vS now advocate as the only correct version) suggests that cooperative breeding has no causal selective consequences for social cognition, but somehow ‘as a side effect… can facilitate performance in socio‐cognitive tasks’ (B&vS, 2016, p. 77). Critically, this perspective is based purely on the putative relaxation of social or energetic constraints associated with cooperative breeding, with no consideration of benefits (see also Dunbar & Shultz, 2007; Tomasello *et al*., 2012). However, if, as B&vS acknowledge, there are no novel cognitive challenges associated with cooperative breeding, then there is no reason to predict that resources freed by reduced constraints should be reallocated preferentially to cognition and brains rather than any other fitness‐enhancing traits.

B&vS outline three components to their hypothesis, which are captured in the following statement: *‘The cooperative breeding hypothesis (CBH) posits that the immediate tasks associated with extensive allomaternal care require motivational proximate mechanisms, such as increased social tolerance or proactive prosociality which, as a side effect, also can facilitate performance in socio‐cognitive tasks. Eventually, over evolutionary time this constellation may also, under specific conditions, facilitate increases in brain size.’* (p. 77). Below, we evaluate the logic and evidence for the three key components of this argument in turn.

## (1) ‘the immediate tasks associated with extensive allomaternal care require motivational proximate mechanisms, such as increased social tolerance or proactive prosociality’

### Logic

In the context of the CBH – a hypothesis about the consequences of *cooperative breeding* – this statement conflates allo*maternal* and allo*parental* care. Allo*maternal* care (which B&vS consider to encompass all care provided by non‐mothers, and so includes *paternal* care) is widespread, particularly in monogamous systems including most birds and some mammals (e.g. some social carnivores, and primates such as owl and titi monkeys; Fernandez‐Duque, Valeggia & Mendoza, 2009; Lukas & Clutton‐Brock, 2013) where fathers as well as mothers contribute to raising young. Cooperative breeding, where *non‐parents* contribute to care (‘*alloparental*’ care), is thought to have frequently evolved from such monogamous systems (Hughes *et al*., 2008; Cornwallis *et al*., 2010; Lukas & Clutton‐Brock, 2012). Critically, B&vS proffer no reason to predict that this allo*parental* care in cooperative breeders should be underpinned by mechanisms other than those already regulating maternal and/or paternal care in monogamous species. Such mechanisms may include, as T&M discussed in the original critique, hormonal priming and responsiveness to signals from young, which may be either active (e.g. begging) or passive (infants’ features themselves act as a signal to solicit care) (for callitrichid examples, see da Silva Mota, Franci & De Sousa, 2006; Barbosa & da Silva Mota, 2013).

### Evidence

#### Increased social tolerance in cooperative breeders

B&vS's argument places strong emphasis on species‐level indices of social tolerance estimated from captive individuals (Burkart *et al*., 2014), but the generalizability and ecological relevance of these findings questionable. Cooperatively breeding species vary widely in group size and structure, degree of reproductive skew and the extent to which skew arises from overt conflict (Cant & Young, 2013; Silk & House, 2016). Levels of ‘social tolerance’ therefore vary widely among cooperative breeders.

#### Increased proactive prosociality in cooperative breeders

The evidence here centres on captive experiments suggesting that callitrichids are more likely than other primate species to perform tasks that directly reward others (Burkart *et al*., 2007, 2014). Putting aside methodological critiques of the experimental design (Thornton & McAuliffe, 2015), these results appear to have little bearing on the CBH's arguments, because they measure behaviour that (1) is not ‘associated with extensive allomaternal care’ (B&vS, 2016, p. 77) and (2) is extremely rare or absent under natural conditions (see below). Whereas prosociality experiments focus on voluntary, unsolicited food donations, largely between adults, the evidence shows that, to quote a paper in which Burkart is a co‐author, food donation in callitrichids under natural conditions occurs ‘almost exclusively from adults to their offspring/[younger] siblings…most sharing events fall under the category of tolerated theft occurring in response to begging…[and] a high percentage of resistance is reported’ (Bullinger *et al*., 2013; see also references cited by T&M). In their response to T&M's critique, B&vS cite a conference abstract to support the claim that 10% of food donations by captive marmosets occur between adults. We are puzzled by this choice of reference as the published abstract does not mention this figure and only describes adults sharing food with young (Martins & Burkart, 2013). More importantly, reports of food transfers among adult callitrichids in the wild are largely restricted to instances of theft by the dominant female (Garber, 1997). Thus, the evidence casts serious doubt on the apparent assumption that cooperative breeding is associated with elevated levels of proactive prosociality.

## (2) ‘…which, as a side effect, also can facilitate performance in socio‐cognitive tasks’

### Logic

B&vS suggest two means by which such a side effect may come about. First, the contributions of helpers may lighten the costs of reproduction for breeding females, allowing them to invest more resources in producing offspring with large brains, which in turn support enhanced cognitive performance (Burkart, Hrdy & Van Schaik, 2009; Burkart & van Schaik, 2010; Isler & van Schaik, 2012). However, this hypothesis (1) provides no explanation for why cooperative breeders should invest these resources in enlarged offspring brains, and (2) evidently does not hold for non‐human primates, where cooperative breeders have unusually *small* brains (Reader & MacDonald, 2002). We return to these issues in section 3.

Second, B&vS suggest that the elevated levels of social tolerance and prosociality they claim are found in cooperative breeders (though see section 1 above) provide a benign social environment in which pre‐existing socio‐cognitive traits can be manifested to a greater degree (Burkart *et al*., 2009; Burkart & van Schaik, 2010). B&vS seem to assume that a reduction in levels of competition and conflict will automatically generate enhanced performance in what they term socio‐cognitive tasks such as social learning and teaching. For instance, they claim that ‘social learning is per definition more efficient than individual learning’ (B&vS, 2016, p. 81) implying that animals would always learn socially if only social circumstances permitted it. This ignores the vast body of literature showing that social learning can be unreliable, generating trade‐offs with more accurate but more costly individual learning (Boyd & Richerson, 1985; Kendal *et al*., 2005; Rieucau & Giraldeau, 2011). Where social learning occurs, it is not simply an emergent product of a tolerant social structure, but a response to particular demands arising from factors such as foraging ecology, predation pressure and resource distribution that affect the *benefits* of social learning (Thornton & Clutton‐Brock, 2011; Smolla *et al*., 2015). Thus, there is no reason to predict that cooperative breeding *per se* (even if it *was* associated with a more benign social environment; see above) should be associated with a higher prevalence of social learning, all other things being equal.

A similar argument holds for teaching. Thornton and colleagues have suggested that the costs of teaching may be reduced in cooperative breeders because they are divided among multiple helpers (Thornton, 2008; Thornton & Raihani, 2008). However, this cost reduction alone cannot explain the emergence of teaching, unless we also consider the benefits. Teaching is expected to evolve where the costs to teachers of promoting learning in pupils are outweighed by the fitness benefits they accrue once pupils have learned. These benefits will be scaled by the utility of the information to be learned: if it is easy to learn through individual or social learning and/or is of relatively low fitness value, the benefits are unlikely to outweigh the costs (Thornton & Raihani, 2008; Fogarty, Strimling & Laland, 2011). This illustrates why the presence of cooperative breeding alone has no explanatory power in understanding the distribution of teaching. For instance, consider meerkats and banded mongooses. Both are cooperatively breeding mongoose species, but the former relies heavily on difficult‐to‐catch and potentially dangerous prey such as scorpions (Thornton & McAuliffe, 2006), whereas the latter predominantly eats slow‐moving or immobile invertebrate prey (Rood, 1975). This explains why there is strong evidence for teaching in meerkats (Thornton & McAuliffe, 2006), but none in banded mongooses.

### Evidence

#### Social learning

B&vS acknowledge that there is no evidence that cooperative breeders show an elevated *ability* to learn socially, but suggest instead that ‘*ceteris paribus* they are more likely to do so’ (B&vS, 2016, p. 80). However, there is no support for this suggestion. The review by Custance, Whiten & Fredman (2002) on which B&vS base their arguments shows no statistically detectable difference in the occurrence of social learning between callitrichids and capuchins in controlled captive experiments (see discussion in T&M). Similarly, Reader's (2003) systematic review found that the prevalence of social learning in wild callitrichids was low compared to capuchins and other social primates. We also note that while capuchins are reported to exhibit social conventions transmitted through social learning (Perry, 2011), no such culturally transmitted forms of social behaviour have been reported in callitrichids.

#### Teaching

The field of animal teaching is in its infancy and more data are needed, but current evidence does not indicate a systematic bias towards cooperative breeders. There is certainly strong experimental evidence for teaching in a number of cooperative breeders (Franks & Richardson, 2006; Thornton & McAuliffe, 2006; Raihani & Ridley, 2008; Colombelli‐Négrel *et al*., 2012), but there is also compelling (albeit observational) evidence from many independent breeders, and a broad survey of all the putative examples of teaching showed no clear biases (Thornton & Raihani, 2008). Among primates, no study has yet demonstrated that knowledgeable individuals act to facilitate learning in others. Further data are needed to establish whether or not some callitrichids teach, but at the moment the weight of evidence is comparable to that for independently breeding macaques (Maestripieri, 1995, 1996; Masataka *et al*., 2009).

## (3) ‘Eventually, over evolutionary time, this constellation may also, under specific conditions, facilitate increases in brain size’

### Logic

This is perhaps the most puzzling aspect of the CBH. As we and others have noted, a release of resource constraints through load‐lightening effects of helpers on breeders’ reproductive effort cannot explain why selection would act to invest those surplus resources in the relative brain size of offspring, rather than any other resource‐constrained fitness‐enhancing trait, such as offspring number or body size, or indeed maternal longevity (Bourke, 2007; Dunbar & Shultz, 2007; Tomasello *et al*., 2012; Thornton & McAuliffe, 2015). Thus, if we are to understand the evolution of increased brain size in humans (one of the central phenomena which the CBH was originally formulated to explain; Burkart *et al*., 2009) we must determine what novel cognitive challenges our ancestors faced (Tomasello *et al*., 2012). Put another way, we need to establish how our ancestors’ ecology affected the benefits of investing differentially in brain size *per se,* rather than the amount of resources available for investment in anything else. As B&vS now clearly acknowledge that cooperative breeding offers no new selective pressures favouring differential investment in brain size, the CBH offers no solution to this problem.

### Evidence

Comparative analyses by Isler & van Schaik (2012) suggest that a higher incidence of allomaternal care (in which they include *paternal* care, and so these findings could be driven as much by biparental care as cooperative breeding) appears to be associated with increased brain size in some mammalian groups. However, it is notable that this relationship *does not hold in primates*, the very group for which B&vS claim the evidence for the CBH is strongest, and which they now suggest may even be the only group in which the hypothesis holds. Indeed, evidence suggests that the evolution of cooperative breeding in primates is linked to greater fecundity (Garber, 1997; Harris *et al*., 2014; Garber *et al*., 2016), not big brains: callitrichids, the only cooperatively breeding primates other than ourselves, have the highest reproductive output of any primates but relatively small brains for their body mass (Reader & MacDonald, 2002).

The lack of any evidence linking cooperative breeding with brain size evolution in non‐human primates might naturally lead one to question the utility of the CBH for explaining large brains in humans. Nevertheless, claims that cooperative breeding could be responsible for our large brains continue unabated (Isler & van Schaik, 2012). B&vS highlight one possible explanation for the lack of supporting evidence; could it be that (other) cooperatively breeding primates simply have not had sufficient evolutionary time to evolve large brains? This seems unlikely, given that the most recent common ancestor of callitrichids and the independently breeding cebids is thought to have lived around 20–22 Mya (Opazo *et al*., 2006), whereas humans and chimpanzees diverged only ~6 Mya (Patterson *et al*., 2006). The picture is similar in birds, where cooperative breeding in the Corvida parvorder emerged around 30 Mya (Edwards & Naeem, 1993), but is not associated with increased brain size (Iwaniuk & Arnold, 2004). The most parsimonious explanation for these patterns would seem to be that cooperative breeding simply has not promoted the evolution of differentially large brains.

## Conclusion

We share with B&vS a strong interest in understanding how and why cognitive traits evolve. However, we remain unconvinced by the logic, assumptions and predictions of the CBH. The lack of clarity regarding the predictions of the CBH is particularly problematic as it leaves it unclear what evidence would be required to falsify it. For instance, does the CBH predict that cooperative breeding should be linked to large brains? If so, isn't it problematic that cooperative breeding does not seem to be linked to large brains? Does it predict increased cognitive ability, or simply improved realized performance? If it predicts changes in ability, why don't cooperative breeders exhibit larger brains or specialized socio‐cognitive mechanisms? If it predicts that cooperative breeders should show improved cognitive performance without improved cognitive ability, then why would the hypothesis predict that they should show the structures or mechanisms (e.g. larger brains, specialized teaching mechanisms) required to improve cognitive abilities? Is the CBH a generalizable hypothesis, or does it just apply to primates? If it just applies to primates, why doesn't it work in primates? Or does it perhaps just apply to humans? If so, why should it just apply to humans, and how are we to test it? To resolve this ambiguity regarding predictions, it seems essential that any attempt to advance this hypothesis be accompanied by a formal mathematical model, grounded in an evolutionary cost‐benefit framework, that generates testable predictions based on explicitly stated assumptions.
